# Parenteral adjuvant potential of recombinant B subunit of *Escherichia coli* heat-labile enterotoxin

**DOI:** 10.1590/0074-02760170133

**Published:** 2017-12

**Authors:** Carlos Eduardo Pouey da Cunha, Clóvis Moreira, Andréa da Silva Ramos Rocha, Paula Fonseca Finger, Carolina Georg Magalhães, Marcos Roberto Alves Ferreira, Odir Antônio Dellagostin, Ângela Nunes Moreira, Fabricio Rochedo Conceição

**Affiliations:** Universidade Federal de Pelotas, Centro de Desenvolvimento Tecnológico, Biotecnologia, Pelotas, RS, Brasil

**Keywords:** aluminium hydroxide, humoral immune response, immune response modulation, rLTB, Mycoplasma hyopneumoniae, P97 adhesin

## Abstract

**BACKGROUND:**

The B subunit of *Escherichia coli* heat-labile enterotoxin (LTB) is a potent mucosal immune adjuvant. However, there is little information about LTB's potential as a parenteral adjuvant.

**OBJECTIVES:**

We aimed at evaluating and better understanding rLTB's potential as a parenteral adjuvant using the fused R1 repeat of *Mycoplasma hyopneumoniae* P97 adhesin as an antigen to characterise the humoral immune response induced by this construct and comparing it to that generated when aluminium hydroxide is used as adjuvant instead.

**METHODS:**

BALB/c mice were immunised intraperitoneally with either rLTBR1 or recombinant R1 adsorbed onto aluminium hydroxide. The levels of systemic anti-rR1 antibodies (total Ig, IgG1, IgG2a, and IgA) were assessed by enzyme-linked immunosorbent assay (ELISA). The ratio of IgG1 and IgG2a was used to characterise a Th1, Th2, or mixed Th1/Th2 immune response.

**FINDINGS:**

Western blot confirmed rR1, either alone or fused to LTB, remained antigenic; anti-cholera toxin ELISA confirmed that LTB retained its activity when expressed in a heterologous system. Mice immunised with the rLTBR1 fusion protein produced approximately twice as much anti-rR1 immunoglobulins as mice vaccinated with rR1 adsorbed onto aluminium hydroxide. Animals vaccinated with either rLTBR1 or rR1 adsorbed onto aluminium hydroxide presented a mixed Th1/Th2 immune response. We speculate this might be a result of rR1 immune modulation rather than adjuvant modulation. Mice immunised with rLTBR1 produced approximately 1.5-fold more serum IgA than animals immunised with rR1 and aluminium hydroxide.

**MAIN CONCLUSIONS:**

The results suggest that rLTB is a more powerful parenteral adjuvant than aluminium hydroxide when administered intraperitoneally as it induced higher antibody titres. Therefore, we recommend that rLTB be considered an alternative adjuvant, even if different administration routes are employed.

Vaccination is the most effective and cost-beneficial way to prevent infectious diseases. The use of recombinant proteins as vaccines has been steadily increasing in recent years. Although such formulations are safer than classic vaccines, recombinant proteins show low immunogenicity due to the lack of pathogen-associated molecular patterns and, therefore, require the use of adjuvants to induce significant immune responses (Dzierzbicka & Kolodziejczyk 2006). Mycobacterial cell wall extracts or whole inactivated mycobacterial cells, saponins, paraffins, oil-in-water emulsions, and aluminium salts are routinely used as adjuvants in research, with the latter two being also used in medicine, and their effects are already well characterised. Most recently, liposomes, immunostimulatory complexes (ISCOMs), viral particles, nanoparticles, CpG, cytokines, and bacterial toxins have been evaluated as potential adjuvants (Williams 2000, Pizza et al. 2001, Yamamoto et al. 2001, Hubbell et al. 2009).

The non-toxic B subunit of *Escherichia coli* heat-labile enterotoxin (LTB) has been explored for use in vaccines because of its capacity to induce a cellular immune response, including cytotoxic T cells (Zhang et al. 2016), and, mainly a strong humoral immune response against antigens that have been co-administered or fused to it (Weltzin et al. 2000, Conceição et al. 2006, Cunha et al. 2014). Furthermore, LTB is a potent mucosal immune response adjuvant (Yamamoto et al. 2001). However, despite these benefits, an understanding of the potential for LTB to be used as a parenteral adjuvant is lacking.

*Mycoplasma hyopneumoniae* is the main etiologic agent of swine enzootic pneumonia, a chronic infection with up to 100% prevalence in pigs. It is spread circulating throughout the world and has been responsible for significant economic losses (Thacker & Minion 2010). The R1 region of the P97 adhesin represents a promising immunogen for use in a recombinant subunit vaccine against this disease (Conceição et al. 2006).

Aluminium salts are the gold standard when it comes to evaluating novel adjuvant molecules because they are routinely used in both human and veterinary vaccines (Brewer 2006, He et al. 2015). In terms of the latter, their main role is to stimulate a strong humoral immune response (Kool et al. 2008). However, adverse reactions are often observed when aluminium-based adjuvants are used (Exley 2016); therefore, the further development of novel alternative safe adjuvants is crucial. The objective of this study was to better understand LTB parenteral adjuvant potential in mice using the R1 repetitive region of *M. hyopeumoniae* P97 adhesin as a model and aluminum hydroxide as the gold standard adjuvant.

## MATERIALS AND METHODS

*Expression, purification, and characterisation of rLTB-R1 and rR1 proteins* - Recombinant LTBR1 and R1 were obtained and characterised as previously described (Conceição et al. 2006). Briefly, LTB and R1 coding sequences were cloned into a pETDEST42™ expression vector (Invitrogen). *E. coli* BL21(DE3) SI was transformed with the constructs, and expression of the recombinant proteins was induced with NaCl (300 mM) and isopropylthio-β-D-galactoside (1 mM IPTG). Recombinant proteins were purified by Ni-NTA affinity chromatography and quantified using the BCA protein assay kit (Pierce).

The antigenicity of recombinant proteins and the ability of rLTBR1 to bind to GM1 were characterised by western blot with anti-R1 monoclonal antibody (MAb) F1B6, and GM1-ELISA, respectively, as previously described (Conceição et al. 2006). F1b6 MAb or rabbit IgG anti-CT were used at 1:3,000 and 1:4,000 dilutions, respectively. Peroxidase-conjugated goat IgG anti-mouse Ig and goat IgG anti-rabbit IgG were used at 1:2,000 and 1:4,000 dilutions, respectively.

*Immunisation of mice* - Six-to eight-week old female BALB/c mice were randomly assigned to three groups of seven animals each. Animals in group 1 were immunised with 50 μg rLTB-R1 (25 μg rLTB; 25 μg rR1). Animals in group 2 were immunised with 25 μg rR1 + 15% Al(OH)_3_. Animals in group 3 were used as a negative control and received a volume of sterile phosphate-buffered saline (PBS) equal to the volume administered to mice in groups 1 and 2. Mice were intraperitoneally (IP) immunised at days 0 and 21. Serum was obtained by centrifuging blood collected from the retro-orbital venous plexus (5 min, 3,000 × *g*).

*Evaluation of the anti-rR1 humoral immune response* - Humoral immune responses were evaluated by indirect ELISA. Maxisorp 96-well polystyrene plates (Nunc) were coated with rR1 diluted in coating buffer (pH 9.6, 400 ng/well at 4°C for 18 h). Plates were washed three times with PBS with 0.5% Tween 20 (PBS-T) between each step. Mice sera were diluted at a ratio of 1:25 in PBS-T and added to plates in triplicate. Horseradish peroxidase (HRP)-conjugated anti-mouse serum (Sig-ma-Aldrich) was diluted at a ratio of 1:2,000 in PBS-T. All reactions occurred in a final volume of 100 μL/well for 1 h at 37°C. Plates were developed with o-phenylenediamine dihydrochloride (OPD) in phosphate-citrate buffer (pH 4.0) for 15 min in the dark, and optical densities were obtained at 450 nm using a plate reader.

Immunoglobulin isotypes IgG1, IgG2a, and IgA were identified using a Mouse Monoclonal Antibody Isotyping Kit (Sigma-Aldrich) according to the manufacturer's instructions. Absorbances were transformed into seroconversion units by dividing the absorbance value of each sample by the mean absorbance of day 0 samples. The mean ratio of IgG1 and IgG2s was used to classify the type of immune response as Th1, Th2, or mixed Th1/Th2.

*Statistical analysis* - Statistically significant differences between samples were determined by ANOVA followed by Tukey's post-test using GraphPad Prism 7. Graphs were created using the same software.

*Ethics statement* - Animal experimentation was approved by the Committee on the Ethics of Animal Experimentation of the Federal University of Pelotas (Permit No. 7722). All relevant national laws and both the university's and national guidelines for animal care and use were followed.

## RESULTS

**Fig. 1:**
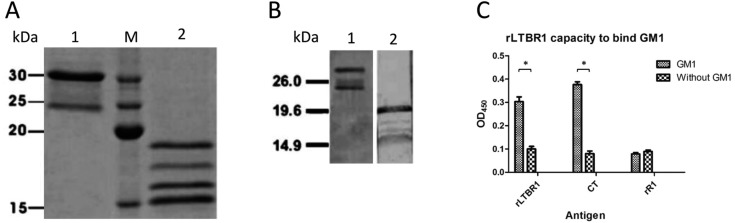
antigenic characterisation of rLTB and rR1 by SDS-PAGE and western blot. (A) SDS-PAGE (15%) of rLTBR1 and rR1 after nickel-affinity chromatography purification. (B) Western blot analysis of rLTBR1 and rR1 with F1B6 monoclonal antibody against R1. In panels A and B, lane 1 is purified rLTBR1, and lane 2 is purified rR1. Benchmarker™ Pre-stained Protein Ladder was used as the molecular weight marker (lane M of panels A and B). (C) The capacity of rLTBR1 to bind to GM1 ganglioside determined by enzyme-linked immunosorbent assay (ELISA). The asterisk indicates significant statistical differences (p < 0.01).

*Production and characterisation of rLTB-R1 and rR1* - Purified recombinant proteins (Fig. 1A) were detected by MAb against the R1 antigen (Fig. 1B), confirming their antigenicity. Purified rLTBR1 was capable of binding bovine GM1 ganglioside in a similar fashion as CT, which was used as a positive control (Fig. 1C). These results confirmed that LTB is biologically active in the rLTBR1 chimera and suggested that LTB may be capable of boosting an immune response.

**Fig. 2:**
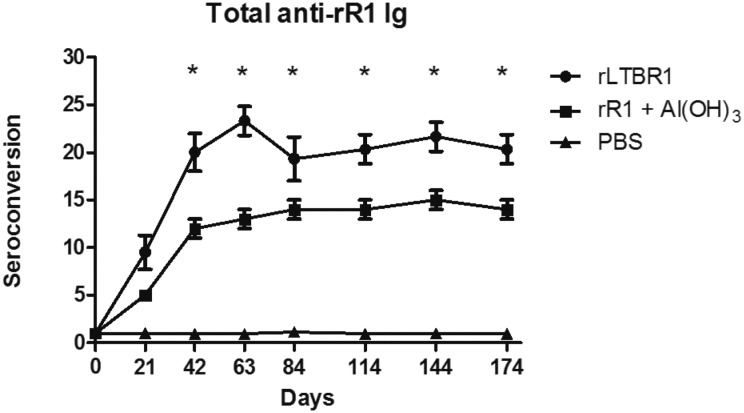
seroconversion of total immunoglobulins against rR1. Indirect enzyme-linked immunosorbent assay (ELISA) was used to assess levels of total immunoglobulins from animals inoculated with either rLTBR1, rR1, or phosphate-buffered saline (PBS). There was a significant statistical difference between animals vaccinated with rLTB or aluminium hydroxide used as adjuvants (represented by an asterisk at different time points, p < 0.05). Seven animals were used per group. Data are shown as mean values with standard deviations from triplicate experiments.

**Fig. 3:**
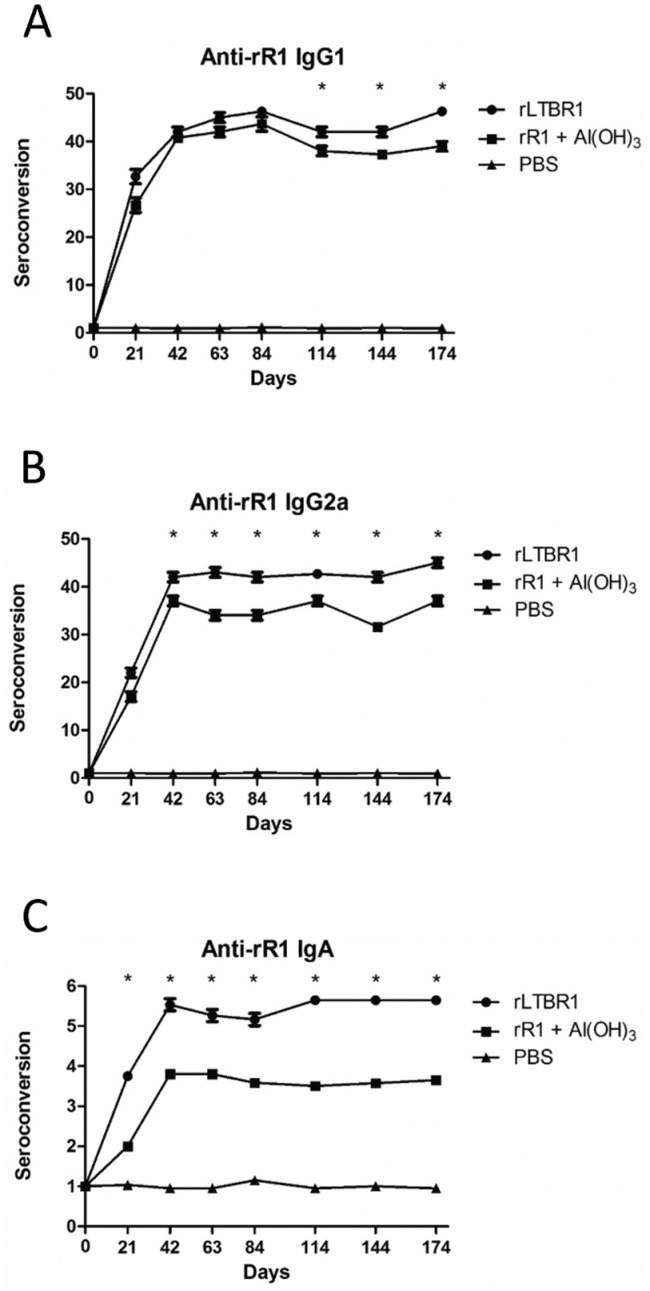
determination of immunoglobulin isotypes in animals vaccinated with either rLTB or aluminium hydroxide as adjuvants. The levels of specific anti rR1 IgG1 (A), IgG2a (B), and IgA (C) were determined by enzyme-linked immunosorbent assay (ELISA) in each experimental group (seven mice per group). Asterisks represent significant statistical differences between groups at the given time points (p < 0.05). Data are shown as mean values with standard deviations from triplicate experiments.

*Humoral anti-R1 immune response* - Animals vaccinated with the recombinant proteins developed a specific humoral immune response against the rR1 antigen (Fig. 2, Fig. 3). Animals inoculated with rLTBR1 showed a higher rate of seroconversion (p < 0.05) for all isotypes than those inoculated with rR1 and aluminium hydroxide (Fig. 3).

**Fig. 4:**
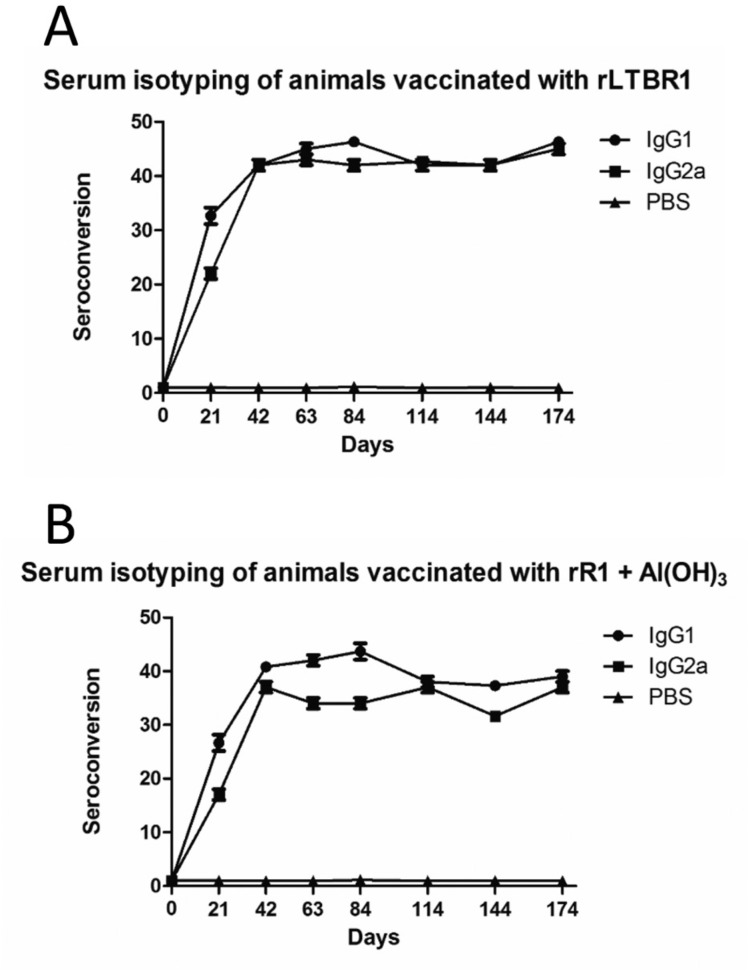
stratification of antibody isotypes by vaccination group. Levels of specific anti-rR1 IgG1 and IgG2a antibody isotypes were compared in animals inoculated with rLTBR1 (A) or rR1 adsorbed to aluminium hydroxide (B) to determine the type of immune response generated by each adjuvant. Seven animals were used per group. Data are shown as mean values with standard deviations from triplicate experiments.

Animals vaccinated with rLTBR1 produced similar levels of IgG1 and IgG2a (with a ratio of 1.42:1 on day 21 and similar levels observed at other time points; Fig. 4A-B), indicating a mixed Th1/Th2 immune response. Animals inoculated with rR1 adsorbed on aluminium hydroxide showed higher levels of IgG1 (with a ratio of 2.1:1 on day 21; Fig. 4A-B), suggesting skewing toward a Th2 immune response at this time point. However, at the remaining time points, the ratio of IgG1 to IgG2a in animals that received aluminium hydroxide was not high enough to suggest a Th2 immune response. Therefore, from day 42 on, the response was considered a mixed Th1/Th2 immune response.

## DISCUSSION

The development of recombinant subunit vaccines has been gaining ground over the past few decades. However, these vaccines show low immunogenicity when compared to attenuated or inactivated vaccines; therefore, the development of adjuvants is crucial (Dzierzbicka & Kolodziejczyk 2006). LTB is a potent adjuvant of the mucosal immune response, which is responsible for conferring protection against a plethora of pathogens (Weltzin et al. 2000, de Haan et al. 2001, Richards et al. 2001, Conceição et al. 2006, Fischer et al. 2010, Cunha et al. 2014, Marchioro et al. 2014, Nandre et al. 2015). However, the parenteral adjuvant effect of rLTB in recombinant protein vaccines has yet to be well characterised.

We evaluated the parenteral adjuvant activity of rLTB by assessing the systemic humoral immune response of mice vaccinated with the recombinant rR1 region of the P97 adhesin of *M. hyopneumoniae.* Our results indicated that rLTB is a stronger parenteral adjuvant than aluminium hydroxide. rLTB induced the production of total systemic immunoglobulins at levels that were twice as high as those induced by aluminium hydroxide. These results agree with those of Rocha and colleagues, who found levels of anti-R1 systemic antibodies that were twice as high as those achieved in response to recombinant BCG expressing LTB (Rocha et al. 2008). Furthermore, both rLTB and aluminium hydroxide extended the humoral immune response against rR1 to at least six months. Taken together, these data, along with the rest of our results, suggest that rLTB should be viewed as a complete adjuvant (de Haan et al. 2001, Conceição et al. 2006, Rocha et al. 2008).

The immune-stimulatory effect of rLTB may be the result of five factors: (1) augmenting antigen presentation by both MHC classes I and II (Nashar et al. 2001, Zhang et al. 2016); (2) activating selective lymphocyte differentiation (Williams 2000); (3) causing dendritic cell activation and maturation (Ji et al. 2015); (4) inducing B7-2 expression on antigen-presenting cells following co-stimulation of CD4^+^ lymphocytes (Yamamoto et al. 2001); and (5) augmenting expression of B lymphocyte activation markers such as MHC class II, B7, CD40, CD25, and ICAM-1 (Nashar et al. 1997).

The IgG1 isotype is generally associated with Th2 humoral immune responses, whereas higher levels of IgG2a indicate a Th1 cellular immune response. Therefore, the ratio of these two isotypes indicates the type of immune response generated against a given antigen (Ferreira et al. 2008). In this study, immunisation of mice with rLTBR1 induced similar levels of IgG1 and IgG2a, suggesting a mixed Th1/Th2 immune response when rLTB is administered intraperitoneally. These findings were similar to those of Rocha et al. (2008), who vaccinated mice with rBCG expressing LTB and R1. However, the results disagree with those of Weltzin et al. (2000) and Richards et al. (2001), who observed strong Th2 responses against different antigens coadministered subcutaneously with rLTB, but Th1 responses when the antigens were administered orally (Weltzin et al. 2000). Likewise, the results of Fischer et al. (2010) and of Conceição et al. (2006) showed distinct types of immune response (Th1, Th2, or mixed) depending on the route of administration of the antigen. Even though it is possible that fusing rLTB to an antigen modulated immune responses, it is equally possible that the antigen itself played a role in modulating these responses. Immunisation with rR1 and aluminium hydroxide induced higher levels of IgG1 on day 21 only, suggesting a Th2 immune response at this time point, which agrees with the findings of several studies that have shown aluminium hydroxide to be an inducer of a Th2 immune response (Brewer et al. 1999, Brewer 2006, Kool et al. 2008). However, from day 42 on, the levels of IgG1 and IgG2a in animals vaccinated with aluminium hydroxide used as an adjuvant were similar, suggesting a mixed Th1/Th2 immune response. Furthermore, BALB/c mice are known to be Th2 responders (Watanabe et al. 2004). Taken together, these results support the hypothesis that rR1 played a role in skewing the response to a mixed Th1/ Th2 immune response, despite aluminium hydroxide typically eliciting and BALB/c mice typically developing a Th2 immune response. Because only studies using R1 as model antigen have shown a mixed Th1/Th2 immune response when LTB was employed as an adjuvant, further animal experimentation is necessary to explore the possibility that LTB is capable of inducing a mixed Th1/Th2 response (i.e., confirming that the results did not reflect R1 modulation alone). It is worth mentioning that the results obtained by Rocha et al. (2008) could reflect modulation of the immune response by BCG, which is known to induce a mixed Th1/ Th2 immune response (Power et al. 1998).

IgA is the main immunoglobulin isotype present in the mucosa, where it plays a primary role, despite also being present in the serum of some species. Immunisation with rLTBR1 induced higher levels of serum IgA than those induced by vaccination with aluminium hydroxide used as an adjuvant. This result indicates that rLTB can induce isotype switching and, therefore, IgA production. This characteristic is already well described for the B subunit of cholera toxin (CTB), which shows 80% amino acid identity with LTB (Millar et al. 2001). Rocha et al. (2008) observed that mice immunised with rBCG expressing LTBR1 presented higher serum IgA levels than mice immunised with rBCG expressing R1 alone. Furthermore, Conceição et al. (2006) showed that rLTB administered via either the intramuscular or intranasal route could induce IgA in the upper respiratory tracts of mice. Of note, the production of IgA seems to be the only aspect of rLTB adjuvant capacity that did note vary depending on the antigen or route of administration used. Taken together, these observations suggest a mechanism through which LTB might augment mucosal immunity: inducing serum IgA production and transport to the mucosal surface. However, further experiments are required to test this hypothesis.

The results of our study suggest that rLTB is a more powerful adjuvant than aluminium hydroxide in terms of the induction of a humoral immune response against the R1 region of *M. hyopneumoniae* P97 adhesin. Therefore, it represents a potential parenteral adjuvant. We believe our results may aid in the development of future vaccines and recommend that rLTB be considered for use as a parenteral molecular adjuvant.
